# Linking Multi-Modal MRI to Clinical Measures of Visual Field Loss After Stroke

**DOI:** 10.3389/fnins.2021.737215

**Published:** 2022-01-05

**Authors:** Anthony Beh, Paul V. McGraw, Ben S. Webb, Denis Schluppeck

**Affiliations:** School of Psychology, University of Nottingham, Nottingham, United Kingdom

**Keywords:** vision, stroke, magnetic resonance imaging, fMRI, DTI, lesion, visual field loss

## Abstract

Loss of vision across large parts of the visual field is a common and devastating complication of cerebral strokes. In the clinic, this loss is quantified by measuring the sensitivity threshold across the field of vision using static perimetry. These methods rely on the ability of the patient to report the presence of lights in particular locations. While perimetry provides important information about the intactness of the visual field, the approach has some shortcomings. For example, it cannot distinguish where in the visual pathway the key processing deficit is located. In contrast, brain imaging can provide important information about anatomy, connectivity, and function of the visual pathway following stroke. In particular, functional magnetic resonance imaging (fMRI) and analysis of population receptive fields (pRF) can reveal mismatches between clinical perimetry and maps of cortical areas that still respond to visual stimuli after stroke. Here, we demonstrate how information from different brain imaging modalities—visual field maps derived from fMRI, lesion definitions from anatomical scans, and white matter tracts from diffusion weighted MRI data—provides a more complete picture of vision loss. For any given location in the visual field, the combination of anatomical and functional information can help identify whether vision loss is due to absence of gray matter tissue or likely due to white matter disconnection from other cortical areas. We present a combined imaging acquisition and visual stimulus protocol, together with a description of the analysis methodology, and apply it to datasets from four stroke survivors with homonymous field loss (two with hemianopia, two with quadrantanopia). For researchers trying to understand recovery of vision after stroke and clinicians seeking to stratify patients into different treatment pathways, this approach combines multiple, convergent sources of data to characterize the extent of the stroke damage. We show that such an approach gives a more comprehensive measure of residual visual capacity—in two particular respects: *which locations in the visual field* should be targeted and *what kind of visual attributes* are most suited for rehabilitation.

## Introduction

Vision loss is a frequent problem following stroke, affecting roughly two-thirds of stroke survivors (Rowe et al., 2013, 2019). Damage to the primary visual cortex is commonly reported (Gray et al., 1989) and usually results in homonymous visual field loss (HVFL)—a complete loss of conscious vision in the contralateral hemifield (Smith, 1962). The scotoma in the visual field can range from a single visual field quadrant (quadrantanopia) to an entire hemifield (hemianopia). Depending on the location of the lesion, the representation of the macular region can be spared—but in some cases it is lost. This impairment greatly impacts quality of life (Papageorgiou et al., 2007), as it affects many aspects of daily living such as reading (Leff et al., 2000), driving (Bowers et al., 2014), and navigating through crowded environments (Goodwin, 2014).

The diagnosis of HVFL in cerebral stroke is typically established using standardized static perimetry. This technique is built on the assumption that areas of the blind field correspond to cortical regions where neurons are damaged to such an extent that they are unable to respond to visual input signaled by the retina. However, it is not clear how useful this measure is for quantifying functional visual capacity and rehabilitation potential. For example, reports of “blindsight”—the ability to respond to visual stimulation in the blind field—suggest that some visual function persists after damage to the visual cortex (Pöppel et al., 1973; Magnussen and Mathiesen, 1989). A recent study by Papanikolaou et al. (2014), using functional magnetic resonance imaging (fMRI), revealed functional responses in V1 corresponding to the “blind” hemifield—apparent cortical sparing that was not captured by static perimetry. This important result suggests that alternative methods of diagnosing the extent of visual capacity is required for individuals with stroke-related HVFL: both, for establishing the details of the visual loss, as well as the rehabilitation potential of patients.

At present, there are no universally accepted, effective rehabilitation programs for HVFL. One promising approach to recovery is perceptual retraining, where repeated stimulation (via training) of specific visual channels is used to induce functional reorganization in the “blind” field (Sahraie et al., 2006; Huxlin et al., 2009). This technique has been widely used in a range of visual deficits not related to stroke, such as amblyopia and age-related macular degeneration (Astle et al., 2011, 2015) and is emerging as an effective tool in rehabilitation. To treat HVFL, different laboratories have implemented perceptual retraining using visual stimuli that are selective for different visual channels, including broadband spatial and temporal frequency patterns and stimuli optimized for motion perception (Pleger et al., 2003; Sahraie et al., 2006; Raninen et al., 2007; Huxlin et al., 2009; Casco et al., 2018; Barbot et al., 2020). However the effectiveness of perceptual training in treating HVFL following stroke appears limited, as improvements tend to vary substantially across individuals. A recent clinical trial using a motion discrimination task for training highlighted this problem, showing no improvements over controls in visual field measures in a large cohort of stroke survivors (Cavanaugh et al., 2020).

**FIGURE 1:**
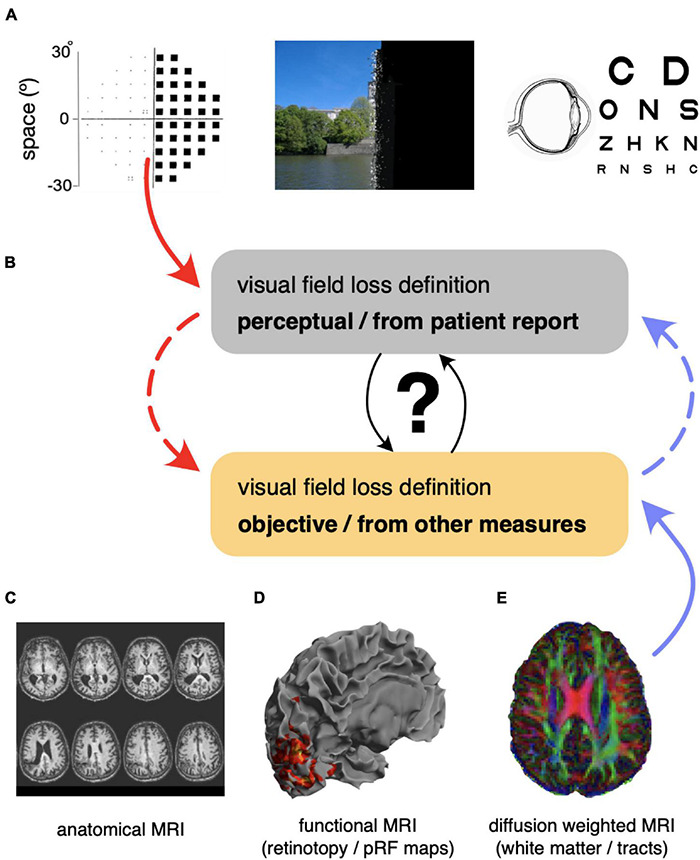
Overview of visual function measurements and how they can be combined. **(A)** Perimetry and informal reports of visual experience give reproducible assessments of patients’ perceptions, but they can be subjective. All participants had a full optometric and ocular health assessment. **(B)** Mapping of standard clinical measurements of visual function to less commonly used assessments of brain function, which provide a complementary picture of visual function loss. **(C)** Example of anatomical lesions as seen with T1-weighted magnetic resonance imaging. **(D)** Functional MRI provides information about visually responsive regions in ipsi- and contra-lesional cortex. **(E)** Diffusion weighted imaging can provide information about the intactness of white-matter pathways underpinning visual function (e.g., optic radiation, OR, vertical occipital fasciculus, VOF).

Although supporting evidence for restitutive approaches to therapy remains weak, this may be because the most appropriate areas of the field are not targeted. A major issue in rehabilitating HVFL is the large individual variability of lesion size, location, time since lesion, and residual visual capacity. Broadly speaking, functional recovery in the “blind” field has been attributed to two possible mechanisms: strengthening of alternative visual pathways or functional reorganization of the spared cortex (Das and Huxlin, 2010). Therefore, the potential for recovery may be limited to individuals with intact cortical structures or alternative visual pathways that could support some level of visual reorganization. The taxonomy of different subtypes of blindsight (Danckert and Rossetti, 2005) further highlights the complexity of how residual *function* in the blind field manifests itself in terms of visual capacity (or even awareness). If therapy could be better guided by functional activity patterns in the brain, it may improve rehabilitation approaches by delineating the visual field locations with the best chance of functional recovery (Figure 1).

Here, we present an analysis of individualized anatomical and functional biomarkers to see how they link to perimetric measures of the visual field. We obtained a full set of optometric measures, including standardized static perimetry, for all participants. To measure the extent of injury caused by stroke, we quantified lesioned and spared cortex in the occipital lobe from anatomical MRI data. To determine the functional integrity of spared visual brain networks, we measured fMRI responses to supra-threshold (retinotopic) visual stimuli presented at sequential points in visual space. This allowed us to use population receptive field (pRF) mapping to reconstruct a representation of visual space across different cortical areas of the visual brain. By estimating the centers and sizes of population receptive fields, we were able to construct a coverage map of the visual field, allowing us to measure the extent of neural responses to visual input inside the scotomatous field defined by perimetry (Dumoulin and Wandell, 2008; Papanikolaou et al., 2014). To identify spared white matter fibers connecting brain areas in visual cortex within the lesioned hemisphere (and between hemispheres), we also acquired diffusion weighted imaging data (DWI) which we analyzed with computational tractography to map out a set of identified tracts underpinning inter- and intra-hemisphere connectivity in occipital, temporal and parietal cortex (Leh et al., 2006; Puig et al., 2017).

We argue that a detailed functional assessment with fMRI will help shed new light on possible targets for rehabilitation in HVFL. Higher-resolution definitions of the patient-specific patterns of residual visual field coverage and cortical integrity across stroke survivors could ultimately improve diagnosis and understanding of the disorder (Millington et al., 2017). While recovery of additional visual field—lost topographic information by damage in early visual areas—is unlikely (Horton et al., 2017), loss maps for higher-level regions that preferentially code e.g., color, motion, or faces may be less affected. In that case subjects may be able to “learn” how to exploit this information in the residual field to support some visual behaviors.

A combination of cross-modal imaging data provides a powerful source of information to characterize the visual capacity of individual stroke survivors with HVFL and supports a personalized medicine approach to stroke intervention. In time, this could help address the marked variability in individual responses to rehabilitation strategies and generate new outcome measures for treatment.

## Materials and Methods

### Participants

Four stroke survivors with HVFL were recruited for this study. A summary of demographic information can be found in Table 1 (and Supplementary Appendix A). We recruited participants through local communities (Nottingham Stroke Research Partnership Group, Nottingham Stroke Club) and advertising to stroke-related organizations (Stroke Association, DifferentStrokes). The inclusion criteria for participation in the study were: (1) Full informed consent, (2) showing symptoms of homonymous visual defect, (3) no evidence of retinal or optic nerve pathology, (4) no manifest strabismus, (5) good ocular motility, (6) absence of spatial neglect, which was assessed through a conventional sub-test of the behavioral inattention test, BIT C (Wilson et al., 2004). All experiments were performed with ethical approval from the School of Psychology ethics committee (F944/F1055R) and all participants gave written, informed consent.

**TABLE 1:** Demographic and basic clinical information about participants.

Participant ID	Sex	Age/years (at the time of scanning)	Visual field defect	Affected side of visual field	Years since stroke	Presence of spatial neglect
11773	F	33	Hemianopia	Right	5	No
13978	M	73	Quadrantanopia	Left	1	No
14196	F	60	Hemianopia	Right	25	No
14326	M	71	Quadrantanopia	Left	3	No

### Vision Tests

A test battery measuring basic visual function and standard ocular health was conducted by a registered optometrist. This included determining (1) existing spectacle prescription, (2) logMAR visual acuity, (3) cover test for ocular alignment, (4) ocular motility, (5) refraction, (6) and an assessment of external (slit-lamp biomicroscopy) and internal ocular health (indirect and direct ophthalmoscopy). The test results for each participant can be found in Supplementary Table 1.

### Static Perimetry

Static perimetry data (for both eyes) were acquired using a standard automated perimeter (M700 Medmount Automated Perimeter). During field testing, a spatially adaptive probe was used, extending out to 50° of visual angle. This allows an initial test pattern of points to be acquired, with additional points added automatically in the region of any suspect field defect; therefore, the number of samples tested depended on the integrity of the subjects’ visual field and ranged between 39 and 168 measurement points per eye. For a particular measurement point, the light intensity required was compared with the age normal value for the participant. When the difference was larger than 6 dB, the neighboring measurement points were automatically added for testing. This method allows for a quick delineation of the visual field using thresholds inside the age normal range (or the edge of the perimeter display). It is worth noting that although this method allows for a large area of the visual field to be covered quickly, subtle defects (or small areas of residual vision) may be missed between test points. One test run (monocular) took around 8 min (longer with increased severity of the visual field loss).

### Fixation Stability and Microperimetry

We used microperimetry to (a) ensure that our field defect measurements from static perimetry were reliable and repeatable and (b) to get a measure of fixation stability. Microperimetry data were collected separately for each eye using a MAIA-2 (iCare, Finland) device. This technique measures retinal sensitivity using the minimum light intensity that patients can perceive when small increments in luminance are used to stimulate discrete locations on the retina. The measurements were performed with a natural pupil in a dim room. Sensitivity was measured using the full threshold 4–2 expert test, with a custom grid of 41 points covering the central 20° of visual angle. Test locations were spaced at 2° over the central 10° of the visual field. The stimulus size was Goldmann III, background luminance was 4 asb (or 1.27 cd/m^2^) and maximum luminance was 1,000 asb (318.3 cd/m^2^) with a 36 dB dynamic range. A red circle, with a size of 1°, was used as a fixation target. Fixation stability during microperimetry was quantified using the Bivariate Contour Ellipse Area (BCEA) measure (Steinman, 1965).

### Magnetic Resonance Imaging

Magnetic resonance imaging data were acquired using a 32-channel head coil on a 3T Phillips Achieva MR system at the Sir Peter Mansfield Imaging Centre (Nottingham, United Kingdom). In each scanning session, we acquired anatomical, diffusion-weighted, and functional data as follows. *Anatomical scans* were acquired using a T1-weighted 3D MPRAGE sequence with the following parameters: 1mm isotropic voxel size, SENSE r = 3, TE = 3.7 ms, TR = 8.13 ms, FA = 8°, TI = 960 ms, FOV 160 × 256 × 256 mm^3^. To aid visualization of lesions, we also acquired T2-weighted images with high inplane resolution (axial) using the following parameters: 0.45 mm inplane voxel size, 3 mm slice thickness, 1 mm gap, TE = 88.9 ms, TR = 3,381 ms, FA = 90°, resulting in images with a matrix size of 512 × 512 × 36.

*Diffusion weighted data* were acquired at 2 mm isotropic resolution, using a single-shot, echo-planar sequence and the following parameters: TE = 57 ms, TR = 8,217 ms, b = 1,000 s/mm^2^. Diffusion weighting was applied in 60 directions, one volume was acquired with b = 0 s/mm^2^. To allow distortion correction using FSL’s *topup* (Andersson et al., 2003; Smith et al., 2004), we acquired two calibration images with reversed phase-encoding directions, resulting in distortions going in opposite directions.

*Functional MRI data* were acquired with a close to axial slice prescription and covered most of the head from frontal to occipital cortex. We used 2D gradient echo EPI, SENSE *r* = 2, TE = 35 ms, TR = 1,500 ms, flip angle = 75°, 24 slices at 3 mm isotropic resolution.

### Anatomical Lesion Segmentation

Semi-automatic lesion segmentation was performed using ITK-SNAP (Yushkevich et al., 2006), using an active contour method. Briefly, we used the following steps to define *lesions masks*: we removed non-brain tissue from the high-solution anatomical images using *optiBET* (Lutkenhoff et al., 2014), which has been optimized for use with brain lesions. In the ITK-SNAP workflow, we applied a threshold to the T1-weighted anatomy image and used seed points inside the lesions to grow using the healthy hemisphere as a frame of reference. To allow direct comparison between healthy and lesioned hemispheres, we made use of left-right reversed images.

### Analysis of Diffusion Weighted Data

We used FMRIB’s Diffusion Toolbox (FDT) to process the diffusion weighted imaging data. Data were corrected for eddy current distortions and subject motion (*eddy);* a fieldmap for correcting susceptibility induced distortions was estimated using *topup.* We used the standard “ball and stick” model implemented in *bedpostx* for estimating the local diffusion parameters and default values with *probtrackx* for probabilistic tractography (Behrens et al., 2003, 2007). In addition, we used *xtract* (De Groot et al., 2013; Warrington et al., 2019) for delineating a subset of tracts proximal to the lesion sites in our participants: left/right: optic radiations, vertical occipital fasciculi, dorsal cingulum, as well as the forceps major.

### Stimulus Presentation

The stimuli presented in our experiments were based on those used in standard retinotopy mapping studies (DeYoe et al., 1996; Dumoulin et al., 2003; Dumoulin and Wandell, 2008). We used the implementation in MGL (Gardner et al., 2018) to present sets of rotating *wedges*, expanding/contracting *rings* and moving *bars* of high-contrast, moving checkerboard stimuli. These stimuli are designed to activate cortical regions representing specific locations of the visual field in a systematic temporal order. Information from the fMRI signal can then be used to reconstruct—for each voxel—the visual field locations that drive its fMRI response.

Participants viewed the stimuli on a BOLDscreen32 (CRS Ltd., Rochester, Kent) at the back of the bore through a mirror mounted on the head coil. Viewing distance to the screen, 119 cm; screen resolution 1440 × 1080 pixels; refresh rate, 100 Hz.

The wedge, ring and bar stimuli had a period of 24 s and to maximize comfort for the participants, we collected data in 6–10 scans lasting five cycles. To help participants maintain fixation and attention during stimulus presentation, we used a simple fixation dimming task (two-interval forced choice): participants had to indicate by button press, which of two intervals contained a darker fixation cross (cyan). Difficulty of the task was adjusted by changing the brightness difference between the intervals in line with a 2-down, 1-up staircase. Participants were trained with the stimulus presentation prior to the scanning session to ensure that they understood the requirements of the task (maintain gaze stability and respond to the color change of the fixation cross).

### Functional Data Analysis

We used population receptive field analysis (Dumoulin and Wandell, 2008) to measure visual field maps in the cortex. Data were analyzed using a combination of FSL (Jenkinson et al., 2012) and custom written software, mrTools (Gardner et al., 2018), running in Matlab (MathWorks, Natick, MA). For methodological details of the setup in Nottingham, see also Xing et al. (2013). We used minimal pre-processing steps, which included motion correction within and across scan repeats (mrTools) and temporal high-pass filtering with a cutoff at 0.01 Hz to remove signal drift.

For each voxel in our fMRI datasets, we estimated best-fit parameters describing a 2D, circularly symmetric Gaussian population receptive field (Dumoulin and Wandell, 2008). Briefly, the model uses a 2D Gaussian profile in visual space (centered at [*x*_0_,*y*_0_], with a standard deviation, σ) to describe the *population receptive field*, the area that integrates the visual stimulus. The predicted fMRI response additionally takes into account temporal delay and blurring by the hemodynamics. For each voxel in our retinotopy scans, we used the measured time series and stimulus-based predictions to compute the best-fit parameters $\left[{\hat{x}}_{0},{\hat{y}}_{0},\hat{\sigma }\right]$ using non-linear least-squares. The quality of the fit was assessed using *r*^2^, the coefficient of determination. A threshold for “reliable” voxel estimates in the visual areas was determined by reference to a non-visually responsive area in prefrontal cortex; we used a value of 3 SD above the mean *r*^2^ in that control ROI, as described in Papanikolaou et al. (2014).

### Mapping to Standard Space

Cortical damage due to stroke can affect image segmentation with tools optimized for neurologically normal brains. In particular, reconstruction of the gray matter and white matter surfaces, inflation and flattening of lesioned hemispheres with *freesurfer* or *caret* may not be routinely possible. To allow the same analysis steps for intact and lesioned hemispheres, we therefore used a volume-based approach to relate anatomical lesions and residual functional responses to the known layout of visual areas. We first registered data from fMRI space to the individual’s T1 anatomy scan (mrAlign, Nestares and Heeger, 2000) and the anatomy scans into MNI152 space (12 dof, FLIRT, Jenkinson et al., 2012). To allow comparisons across imaging modalities, we used the 1mm isotropic standard MNI space as a common target for final visualizations and data summaries. For example, statistical maps derived from BOLD fMRI (pRF maps) were super-sampled from 3 mm isotropic into this 1 mm^1^ space. This was particularly important, as it allowed us to map fMRI responses, anatomical lesion quantification and other derived measures, e.g., from the pRF analysis, to be characterized by the same probabilistic atlas (viz Wang et al., 2015). We used the maximum probability maps derived from the volume based analysis of the Wang et al. (2015) atlas to characterize the different visual regions.

### Visual Field Coverage in Intact and Lesioned Hemispheres

To show residual visual responses in cortex, we used two approaches: (1) Plots of the pRF centers $\left[{\hat{x}}_{0},{\hat{y}}_{0}\right]$ super-imposed on the corresponding static perimetry results. (2) Visual field coverage maps that visualize the integrated area covered by pRFs for a given region of interest (Papanikolaou et al., 2014). Relating the perimetry data to the cortical visual responses elicited in the fMRI experiment allowed us to look for mismatches between the two techniques, in particular fMRI responses to stimuli in areas declared non-functional by standard perimetry. The visual field coverage maps can be constructed from pRFs found in specific ROIs, highlighting visual field locations that are being driven by activity in those cortical regions.

## Results

### Homonymous Visual Field Defects

**FIGURE 2:**
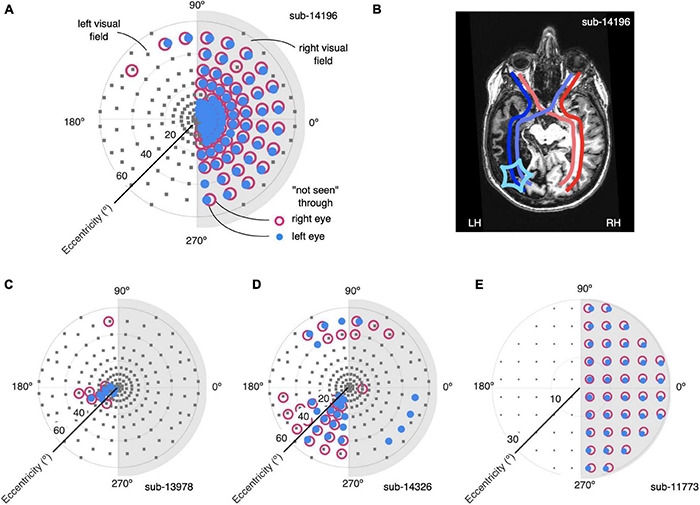
Definition of homonymous visual field loss with perimetry and correspondence to anatomical lesions. **(A)** Visual field definition by standard perimetry for participant 14196. Test locations (gray squares) were equally spaced across the left and right portion (light gray shading) visual field. Locations at which the participant did not respond to stimuli are shown for both monocular tests [red open symbols, tested through right eye (OD), close blue symbols, tested through left eye (OS)]. There is a tight correspondence between the two sets of measurements, indicating a homonymous visual field defect. **(B)** Annotated slice of anatomical MRI scan for participant 14196. Dark blue, red lines show projections from the temporal retinae. Light blue, red lines show projections from the nasal retinae. The extent and location of the cortical lesion (star symbol in left hemisphere, LH) determines the contralateral visual field loss. There was no apparent cortical loss in the right hemisphere, RH, reflecting the perimetry measurements in **(A)**. **(C–E)** Perimetry results for the other participants in our study in the same convention as **(A)**. Note that for participant 11773 perimetry was performed with slightly different parameters and only extended to 30° eccentricity.

The static perimetry data revealed homonymous visual field defects across all four stroke survivors (Figures 2A,C–E), two with complete right sided hemianopia with no apparent macular sparing (Figures 2A,E), two with partial or complete lower left field quadrantanopia (Figures 2B,C). The estimated pattern defect and mean deviations were consistent across both eyes in all subjects, as seen in Table 2 (sensitivity measurements across the visual field for each participant can be found in Supplementary Appendix B). Furthermore, these results were consistent with microperimetry data.

**TABLE 2:** Perimetry results (standard clinical scores).

Participant ID	Visual field defect	Pattern defect (dB)		Mean deviation (dB)	
		Left eye	Right eye	Left eye	Right eye
11773	Hemianopia	17.42	18.71	–20.31	–14.59
13978	Quadrantanopia	7.34	9.11	0.04	–0.12
14196	Hemianopia	22.01	21.22	3.89	3.74
14326	Quadrantanopia	12.39	11.02	2.53	2.71

*Statistical summary of static perimetry results. The pattern defect is based on spatial correlation, measuring the clustering and depth of the defect. The overall defect, taken as the mean difference between the age normal hill of vision (HoV) and each participant’s HoV.*

### Fixation Stability

To be able to measure reliable results with retinotopic fMRI stimuli, it was important to establish that the participants were able to maintain stable fixation at the center of the screen. During microperimetry, we measured the BCEA containing 63% of fixations, a widely used evaluation of fixation stability (Crossland et al., 2004). Participants 11773, 14196 and 14326 showed relatively small BCEA values, ranging from 0.4°^2^ to 1.7°^2^, whereas participant 13978 had a large BCEA (left eye: 17.9°^2^, right eye: 26.9°^2^) indicating more unstable fixation.

### Anatomical Lesions Are Highly Variable

**FIGURE 3:**
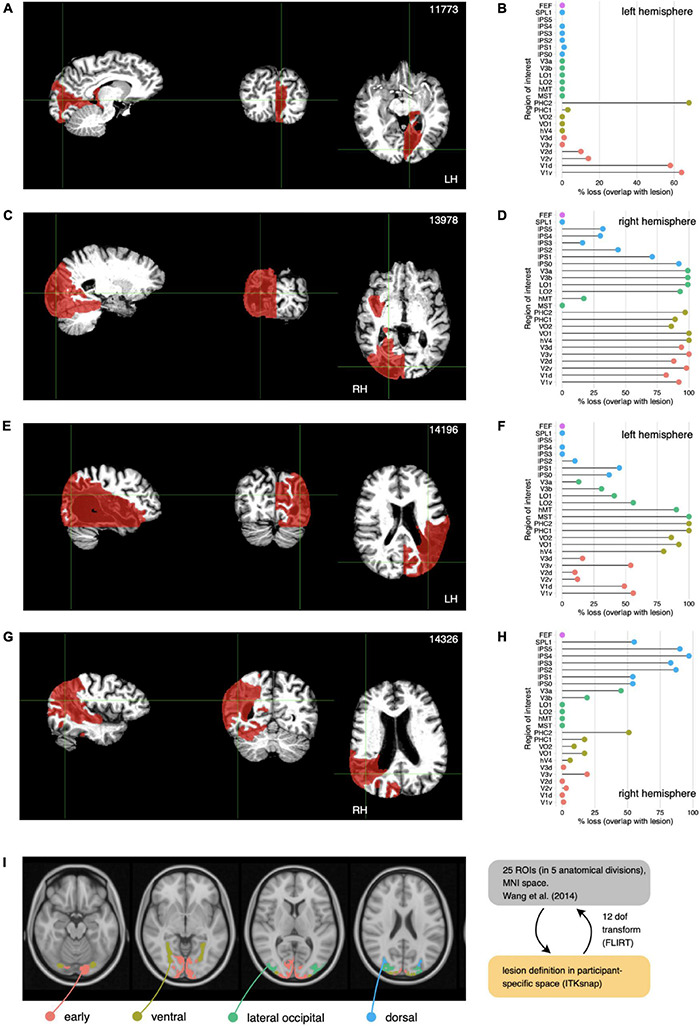
Anatomical lesions. Locations and quantification for each participant. **(A,C,E,G)** Brain-extracted anatomical images in sagittal, coronal, and axial view (grayscale), in planes that show the extent of the anatomically defined lesions (red). We used *optiBET* (Lutkenhoff et al., 2014) for skull stripping. **(B,D,F,H)** Lesion damage by subregions. To quantify lesions with respect to known visual areas, we transformed the lesion masks in participant space into MNI space and computed percentage overlap with the regions of interest defined in the probabilistic atlas of Wang et al. (2015). Each row (dot) in the dotplot shows percentage loss for an ROI (V1 bottom, to FEF, top). To aid visualization, we grouped ROIs into cortical territories: early visual (red), ventral (dark yellow), lateral occipital (green), dorsal (blue), see also Figure 5. **(I)** Location of cortical ROIs from Wang et al. (2015) atlas. The location of the four cortical territories (colors) comprising 25 ROIs are shown on axial slices of the MNI152 brain (gray). Note that the Wang et al. (2015) atlas also included labels for frontal eyefiels (FEF), but none of the participants showed damage in that region.

Both the anatomically defined lesion masks and the T1-weighted anatomical images and brain-extracted derivatives were normalized into standard space (Figure 3). To quantify the lesions, we computed the percentage overlap between the lesions definitions in individual participants and the 25 cortical ROIs defined by the probabilistic atlas of Wang et al. (2015). To aid visualization, we grouped the ROIs into 5 cortical territories: *early visual* (V1v, V1d, V2v, V2d, V3v, V3d), *ventral* (hV4, VO1, VO2, PHC1, PHC2), *lateral occipital* (MST, hMT, LO1, LO2, V3a, V3b), *dorsal* (IPS1, IPS2, IPS3, IPS4, IPS5, SPL1) and *frontal* (FEF). The dot plots in Figures 3B,D,F,H indicate the proportion of these areas lost, 0% indicating total sparing of cortex in the corresponding region of interest, and 100% indicating complete loss.

#### Lesion Size Is Not a Good Indicator of Visual Field Loss

All participants showed damage to early and ventral regions, but the extent and pattern of the damage varied widely across participants. For example, participant 11773—with hemianopia—had stroke lesions that were only co-located with early visual areas (Figure 3A). Conversely, participant 13978—with quadrantanopia and an ostensibly smaller visual field loss—showed a much larger anatomical lesion (compare Figures 2C, 3C).

#### Homonymous Visual Field Defects Are Typically Characterized by Damage to the Early Visual Cortex (V1–V3)

We observed this pattern in three of the four participants (11773, 13978, and 14196). In one participant (14326), the lesion site was dominated by lateral occipital regions.

### Diffusion-Weighted Imaging Indicates Tract-Level Damage

To assess white matter integrity at the level of major identified tracts, we used diffusion-weighted imaging and probabilistic tractography. The most relevant tracts for the stroke survivors in this study were those connecting areas within and between the occipital lobes, namely the *vertical occipital fasciculi (vof, left and right), the optic radiations (or, left and right) and the dorsal bundles of the cingulum (cbd, left and right).* In addition to these lateralized structures, we also analyzed the *forceps major*, a large bundle connecting the visual areas via the splenium of the corpus callosum.

**FIGURE 4:**
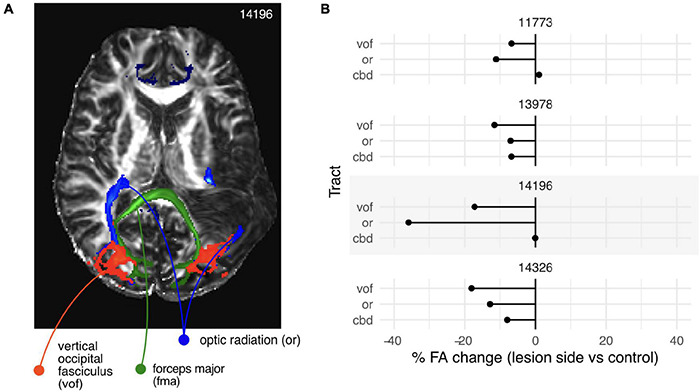
White matter intactness, results from diffusion weighted imaging and analysis. **(A)** White matter tracts identified from diffusion weighted data by probabilistic tractography (*probtrackx).* Blue, *optic radiations* (left and right); red, *vertical occipital fasciculi* (left and right); green, *forceps major*, a large fiber bundle of the corpus callosum connecting visual areas in the left and right hemisphere. Grayscale image, map of fractional anisotropy. **(B)** Percentage change in fractional anisotropy between the lesioned and non-lesioned hemisphere for *vof, or*, and *cbd.* For all participants (sub-panels), there was a clear reduction in the FA values in the lesioned hemisphere up to 40%, although the magnitude and pattern of reduction was markedly different across participants.

After computing the microstructural measures using a diffusion tensor fit (*dtifit*), and probabilistic tractography (*bedpostx*) we used an automated method for defining the tracts of interest (*xtract*, see section “Materials and Methods” for details). Figure 4A shows a rendering of those tracts superimposed on the fractional anisotropy (FA) map for participant 14196 (right hemianopia). An extended area of reduced FA is clearly visible in the left hemisphere (compared to the right). This area corresponds to part of the stroke lesion.

Despite the stroke lesion overlapping the optic radiation (OR) in the left hemisphere, the probabilistic tractography identified tracts on both sides. It is worth noting that although the left OR looks completely disrupted in the particular section shown (blue label, Figure 4A), a bundle was still present, although of a reduced volume: in this participant it measured 8,470 mm^3^ in the left hemisphere compared to 12,254 mm^3^ in the right (using the default threshold of 0.001 on the tract probability maps).

To quantify the differences in the underlying microstructure, we computed the mean FA value in the identified tracts and compared the values in the lesioned hemispheres to those in the non-lesioned ones. For all participants, there was a clear reduction in the FA values in the lesioned hemisphere, although the magnitude and pattern of reduction was markedly different across participants (Figure 4B).

### Visual Field Maps Reveal Cortical Responses in “Blind” Portions of Visual Field

**FIGURE 5:**
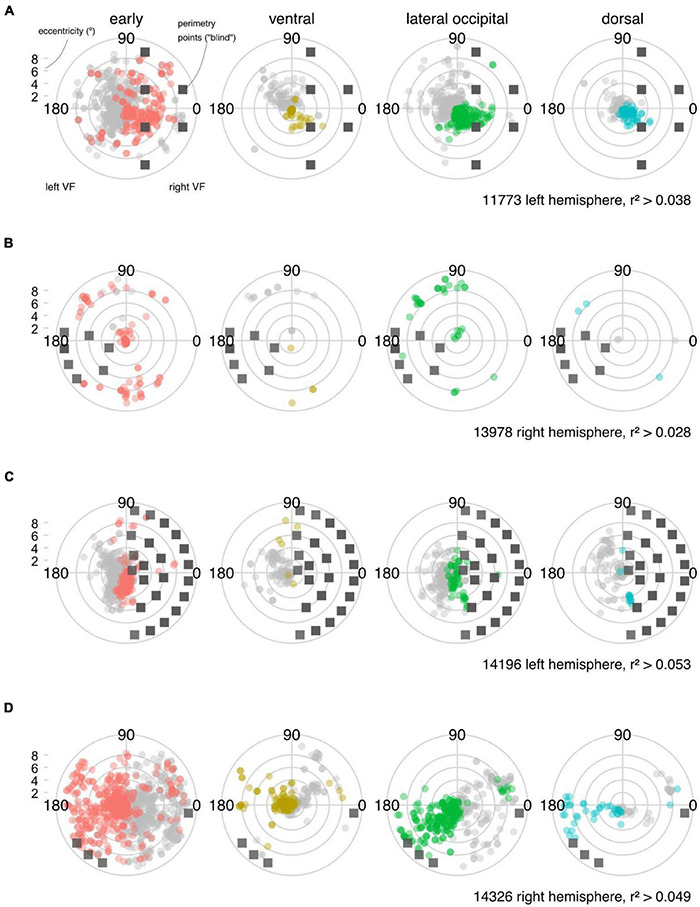
Visual field maps derived from functional MRI and population receptive field (pRF) analysis. **(A–D)** Population receptive field (pRF) centers are plotted in polar coordinates for each participant to show the voxel representations in the visual field containing the scotoma. Note that in this plot, we only consider the centers of the pRFs that exceed an r^2^ threshold determined in a control region. The scatter plot of pRF centers (colored symbols) is overlaid on the scotoma definition from perimetry (gray squares). Data for the ispi-lesional visual field (from the unaffected hemisphere are not shown in light gray symbols). Note that for participant 13978, fixation stability was an issue (and even with gaze-contingent microperimetry, acquiring robust data was challenging). For participant 14326, robust macular sparing was apparent in both standard and microperimetry. Despite this, the fMRI measurements still reveal a different pattern of loss (cf Table 3 and Supplementary Figures 1, 2).

To identify any residual functional activity in the regions of cortex corresponding to visual field defects, we mapped the pRF centers $\left[{\hat{x}}_{0},{\hat{y}}_{0}\right]$ from the lesioned hemisphere onto the corresponding static perimetry results as seen in Figure 5. As in the visualization of the anatomical results, we grouped the pRF models into cortical territories (*early visual, ventral, lateral occipital* and *dorsal regions*. [The Wang et al. (2015) atlas also included a frontal region of interest, but no pRF model fits exceeded the threshold for reliable responses.]

**FIGURE 6:**
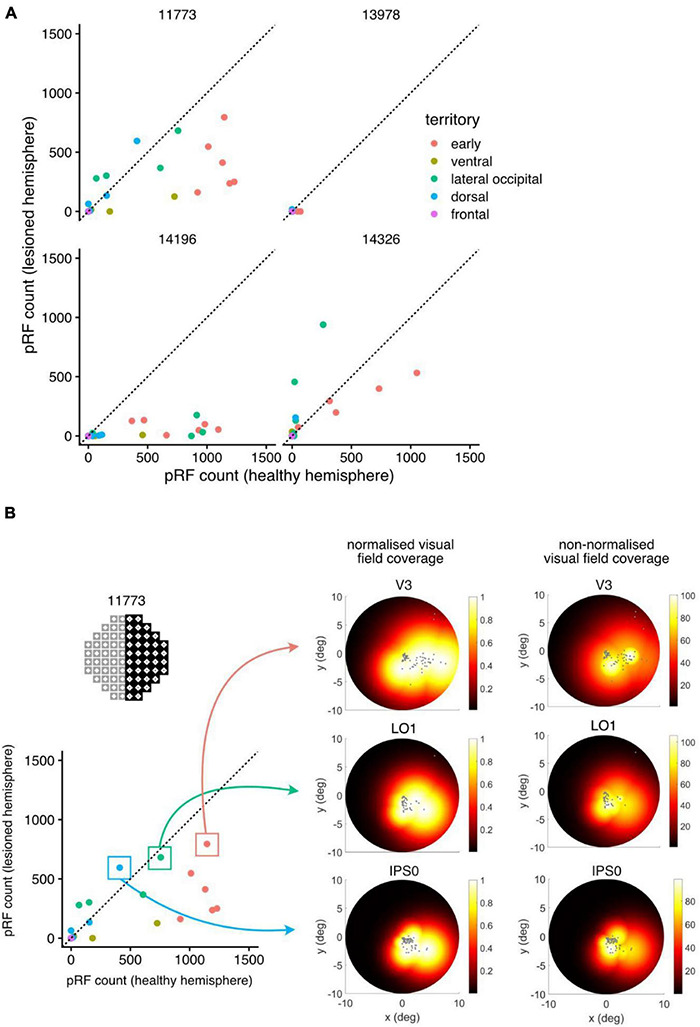
Quantifying and visualizing residual function from population receptive fields (pRF). **(A)** Comparing population receptive field (pRF) results between the lesioned and healthy hemisphere (participant 11773). For each ROI, the number of pRFs (approximated as circles with radius σ) that intersect with the convex hull of the scotoma was calculated. As a control, pRFs intersecting with the scotoma flipped into the non-affected field were counted—this corrects for changes in size of ROIs as well as the ability of the pRF model to fit responses in higher visual areas. Counts related to voxels in 1 mm standard space, so equivalent units are mm^3^. **(B)** Three ROIs with high pRF counts in the stroke hemisphere; the pRFs corresponding to voxels in this regions were used to construct normalized and non-normalized visual field coverage maps (cf Papanikolaou et al., 2014). The domain of the color map used for the non-normalized versions here spanned zero to the maximum pRF amplitude over all voxels. The visual field coverage maps across all three identified subregions show consistent coverage across the lower quadrant.

To quantify the visual capacity in the “blind” region, we took into account the extent of each voxel pRF (circular region in visual space, centered at $\left[{\hat{x}}_{0},{\hat{y}}_{0}\right]$ with radius $\hat{\sigma }$) and measured the number of pRFs that intersected with the scotoma. As the absolute count of reliable pRFs can also be affected by changes in signal-to-noise-ratio of the BOLD signal across participants, we compared these counts to those in the non-lesioned hemisphere. By flipping the “blind” region in the perimetry across the vertical meridian (y-axis) and measuring the number of intersecting pRFs for the non-lesioned hemisphere, we therefore obtained a directly comparable count (within participant and unaffected by changes in pRF fit quality across ROIs; Figure 6). The scatter plots in Figure 6A show the number of pRFs intersecting with the blind field in the lesioned hemisphere compared to those in the non-lesioned hemisphere. ROIs that fall close to the diagonal line in these plots represent similar pRF counts in the stroke and healthy hemisphere—indicating unaffected levels of visual response for that region. Points that fall well below the diagonal identify ROIs whose response was substantially reduced in the lesioned hemisphere.

We used a non-parametric statistical test to quantify differences in the distribution of pRF responses overlapping with scotoma and corresponding portion in the intact visual field. For each ROI in each participant, we obtained the distributions of *r*^2^-values and compared them using a Kolmogorov-Smirnov test. Only pRF responses from uniquely identified functional voxels were used for this test. Table 3 shows the D statistic and corresponding *p*-values. The null hypothesis, H_0_, for this test is that *r*^2^-values in the healthy/stroke ROIs come from the same distribution. For nearly all ROIs, the distributions in healthy and stroke hemispheres were significantly different from each other based on this test. For some ROI, notably outside early visual cortex, there were no pRFs that overlapped with either the scotoma or the corresponding region in the intact visual field. This could less consistent alignment of these regions across participants (and with the probabilistic atlas).

**TABLE 3:** Differences in population receptive field coverage in lesioned and healthy hemispheres.

ROI	11773		13978		14196		14326	
	*D*	*p*	*D*	*p*	*D*	*p*	*D*	*p*
V1v	0.568	<1e-8	0.659	<1e-8	0.627	<1e-8	0.341	<1e-8
V1d	0.587	<1e-8	0.396	<1e-8	0.73	<1e-8	0.419	<1e-8
V2v	0.777	<1e-8	0.546	<1e-8	0.859	<1e-8	0.546	N.S
V2d	0.377	<1e-8	0.316	<1e-8	0.73	<1e-8	0.206	<1e-8
V3v	0.806	<1e-8	0.888	<1e-8	0.916	<1e-8	0.842	<1e-8
V3d	0.352	<1e-8	0.536	**N.S**	0.783	<1e-8	0.130	<0.01
hV4	0.709	<1e-8	1.000	<0.01	0.788	<1e-8	1.000	<0.01
VO1	0.862	<1e-8	0.763	<1e-5	0.826	<1e-8	–	–
VO2	0.41	<1e-8	0.784	<1e-8	0.676	<1e-8	0.907	<1e-8
PHC1	0.38	<0.01	0.969	<1e-8	0.352	**N.S**	–	–
PHC2	0.862	<1e-8	–	–	0.641	<1e-8	0.597	<1e-5
hMT	0.526	<1e-8	1.000	<1e-8	0.594	<1e-5	0.652	<0.01
LO1	0.213	<1e-8	0.636	<1e-8	0.626	<1e-8	0.732	<1e-8
LO2	0.271	<1e-8	0.966	<1e-8	0.451	<1e-8	-	-
V3a	0.207	<1e-8	0.622	<1e-8	0.902	<1e-8	0.623	<1e-8
V3b	0.152	<0.01	0.515	<0.01	0.789	<1e-8	0.585	<0.01
IPS0	0.143	<1e-5	0.305	<0.01	0.379	<1e-8	0.932	<1e-8
IPS1	0.211	<1e-5	0.750	<1e-5	0.444	<1e-5	0.929	<1e-8
IPS2	0.675	<1e-8	–	–	–	–	–	–
IPS3	0.608	<0.01	–	–	–	–	–	–
IPS4	0.750	**N.S**	–	–	–	–	–	–
SPL1	0.500	**N.S**	–	–	–	–	–	–
FEF	1.000	**N.S**	–	–	–	–	–	–

*Statistical summary of regions of interest whose pRF responses overlap with scotoma (voxels from lesioned hemisphere) or corresponding portion in intact visual field from intact hemisphere). We compared the distributions of r^2^-values of the healthy and stroke ROIs using a Kolmogorov-Smirnov test. Only pRF responses from uniquely identified functional voxels were used. Rows, regions of interest identified from probabilistic atlas. Columns, D statistic and corresponding p-values for each ROI in each participant. The null hypothesis, H_0_, for this test is that r^2^-values in the healthy/stroke ROIs come from the same distribution. N.S., not significant at a = 0.01. Dashes denote ROIs for which there were no overlapping pRF responses in either the healthy or stroke hemispheres.*

In both participant 11773 (hemianopia) and participant 14326 (quadrantanopia), we were able to identify ROIs that had near normal counts of reliable pRFs, indicating visual activity in the “blind” region of the visual field. Interestingly, such responses were more prominent in *early visual* and *lateral occipital* regions of the lesioned hemisphere. Overall, participant 14196 (hemianopia) showed a smaller extent of functional activity within the “blind” region, as seen in Figures 5C, 6A. Participant 13978 (quadrantanopia) showed little evidence for any residual function in regions corresponding to the scotoma.

To demonstrate how these spared cortical regions are represented in the visual field, we constructed the normalized visual field coverage maps for three selected ROIs (*V3, LO1, IPS0*) in participant 11773 (for methods refer to Papanikolaou et al., 2014). These regions showed a high number of pRFs represented within the scotoma (these ROIs fell close to the diagonal line in the scatter plot in Figure 6B). The visual field coverage maps derived from pRFs in these regions showed residual functional activity in the right, lower quadrant of the hemifield within the scotoma. This consistency across visual field coverage maps, derived from different regions, adds supporting evidence for some common residual processing of information from these regions of the visual field.

## Discussion

This paper demonstrates a multi-modal approach using brain imaging (MRI) to carefully characterize the link between perimetry-derived visual field loss and biological markers of stroke damage. We used lesion definitions based on anatomical scans, measures of white matter integrity from diffusion imaging and responses to visual stimuli from functional MRI in four stroke survivors with homonymous visual field defects. In three out of the four stroke survivors, we showed strong evidence of residual functional activity in parts of the visual brain representing visual field locations defined as scotomatous by perimetry. Our results are consistent, both with reports of blindsight (Pöppel et al., 1973; Magnussen and Mathiesen, 1989) and the findings by Papanikolaou et al. (2014), who used fMRI to map out spared visual cortex responses in four stroke survivors with quadrantanopia.

### Mismatches Between Perimetry and Imaging Defined Responses

It is useful to consider the mismatches between the clinical, static perimetry and imaging defined measures in turn: to (a) lesion definitions from anatomy scans, (b) fMRI derived population receptive field (pRF) maps, (c) any damage in diffusion imaging defined white matter tracts. Each of these measures provides complementary information about the stroke-related damage in the brain. Ultimately, building up a more detailed, “multivariate” picture of stroke lesions in this way will help identify new strategies for rehabilitation.

#### Perimetry and Anatomical MRI

The pattern and extent of cerebral lesions varies substantially across stroke survivors, even in situations where the perimetry-defined visual field loss is remarkably similar. For example, participants 11773 and 14196 in our study, both with hemianopia, have very distinct and different patterns of cerebral lesions, yet a nearly indistinguishable visual field loss. This in itself underscores the value of additional personalized measurements to characterize the patient-specific visual field loss.

Many reports of anatomical lesion definitions of HVFL provide only a cursory outline of the lesion location, pointing to the occipital cortex or the occipital pole (Fujino et al., 1986; Sanchez-Lopez et al., 2020). However, there is a drive for personalized approaches to treatment and constant improvements in imaging technology are likely to facilitate this (Hinman et al., 2016).

In the current study, we used information from a probabilistic atlas of visual areas (Wang et al., 2015) to further subdivide the cortical parts of the stroke lesions. Other cortical atlases and parcellations may provide additional information (van Essen et al., 2019), but our choice of using a probabilistic atlas of visual areas was driven by a similarity in the methodology used to define topographically organized areas, and a growing literature on the functional properties of these areas (Wandell et al., 2007). The definitions of the 25 regions in each hemisphere, alongside which areas are most and least affected by the stroke, suggest particular stimulus categories or “channels” that may be most useful for rehabilitation.

#### Perimetry and Visual Responses in Functional MRI

We found residual visual responses to stimuli presented inside the scotoma of all participants. In three out of the four stroke survivors, residual functional activity was robust and in one case (participant 14326) the functional responses were broadly similar to the non-lesioned hemisphere (see Figures 5D, 6A). For one participant (13978, Figures 5B, 6A) this pattern was much less clear, but we note that this individual had particularly unstable fixation, as seen in the microperimetry derived measure of fixation stability (BCEA). This has the potential to reduce the reliability of functional maps and in situations where fixation stability is poor, some form of gaze contingent mapping may be beneficial. At present, we do not know whether ocular instability has masked residual cortical activity within the defined region of the scotoma.

#### Perimetry and Diffusion Weighted Imaging/Tractography

We obtained diffusion weighted imaging data for all participants in our study. By fitting a diffusion tensor model, these data can be used to compute voxel-wise statistics such as *fractional anisotropy* (FA) and *mean diffusivity* (MD) that are sensitive to changes in microstructure. Without much further analysis, these images provide an additional image contrast that can be useful in clearly defining lesion damage (Tae et al., 2018). In addition, we performed probabilistic tractography using tools from FDT (*bedpostx, probtrackx, xtract).* For each participant, we identified the following tracts in the posterior part of the brain: left and right *optic radiations*, left and right *vertical occipital fasciculi*, *forceps major* (connecting visual areas in the left and right hemispheres) as well as the posterior portion of the *cingulum*.

### Potential Sites for Rehabilitation and Optimal Stimuli for Perceptual Learning

The use of perceptual learning paradigms rely on the individual’s capacity to relay relevant information about the stimulus from the retina to visual cortex (Horton et al., 2017). If the cortex, or the connecting white matter tracts are completely lost, any changes in visual function, or recovery in the scotoma cannot be expected. Therefore, to maximize potential success of any rehabilitation approach it is important to identify strategies that are consistent with individual physiology. In order to do this, two pieces of information are crucial: (1) the *locations* of any sparing in the visual field and (2) which functional/anatomical regions are partially or fully intact. This information could be used to guide a personalized approach to *where* in the visual field training should occur, say, a patch extending from the fovea to a known eccentricity in the lower left quadrant, which was identified during fMRI mapping.

Importantly, the information about spared anatomical regions (or even functional subdivisions) suggests a class of stimuli or tasks that might provide the largest responses, and therefore the most likely route to rehabilitation. If we consider the field loss revealed by perimetry in terms of topographically mapped regions, then the use of imaging allows the measurement of other “down-stream” loss maps in the post-stroke brain, which may look quite different. Recovery of lost topographic information due to damage in early visual cortex is less plausible (Horton et al., 2017), but if the loss map for regions that prefer e.g., color, motion, or faces are less affected, then subjects may be able to “learn” how to exploit this information in the residual field to support visual behaviors. In some ways, this is akin to the problems introduced by retinal or cortical implants for vision restoration. In that situation, the device introduces distortions and lossy information about the image, but patients can re-learn to use this new input for a range of visual behaviors (for discussion see Beyeler et al., 2017).

For example, if ventral regions are relatively spared compared to dorsal regions, then a training program based on objects, faces, and other stimuli preferentially processed in these regions suggests itself (Kanwisher et al., 1997; Grill-Spector, 2003). The location for visual rehabilitation in the affected visual field should be guided by where (functional) responses can be elicited and then further enhanced by training. This may include areas of the visual field with “functional MRI-defined blindsight”—declared by perimetry to be non-functioning but responding in the fMRI experiment. Additionally, they may also include a more fine-grained definition of areas appearing as *spared* in perimetry, but not well-defined due to the relatively coarse spatial scale of perimetry.

By way of example, consider the imaging results from participant 11773 (right hemianopia). Residual functional activity within the scotoma, measured across different visual areas, represents the lower right quadrant of the visual field. We propose that restitutive approaches should target this location for perceptual training. The ROIs labeled as contributing to this residual function point toward the class of stimuli that might be most suitable. For example, there is good evidence that *V3* is sensitive to chromatic- and luminance-defined motion stimuli (Gegenfurtner et al., 1997; McKeefry et al., 2010). To recruit this part of cortex, therefore, training using moving chromatic and luminance grating stimuli may be most appropriate.

This principle could be applied for other ROIs identified in this way, such as *LO1* and *IPS0*, though visual pRFs representing the scotoma that were defined with standard retinotopy stimuli may be less efficient at driving higher level regions differentially. In addition, the distribution of residual function may vary substantially among other stroke survivors. An interesting question for future work would be to test whether the least complex stimuli consistent with residual function (such as gratings, curvature defined stimuli) or more complex stimuli (such as faces, objects, etc.) are stronger drivers for rehabilitation.

Restitutive approaches are perceptually challenging and require a significant time commitment from stroke survivors—often long hours training on a computer display are required. To ensure the best chance of success and to improve training compliance, it’s important to establish a protocol that is guided by the individual functional activity pattern, using stimuli that the patient’s visual brain can learn to respond to.

## Conclusion

Multi-modal imaging in stroke survivors provides informative data on both the lesion and spared functional regions in visual cortex for a relatively small time commitment and cost. The scanning protocol used here took only 1 h for data acquisition. We believe our approach could particularly inform perceptual learning-based rehabilitation, by enabling the targeting of specific visual field locations and selecting the most optimal class of stimuli. Our work shows that different individuals might benefit from rehabilitation that targets a specific set of downstream cortical regions. Crucially, detailed mapping in this way could also serve to inform clinicians to direct stroke survivors to other rehabilitation approaches, if imaging-based mapping reveals that no residual function is measurable across the majority of visual areas in the lesioned hemisphere.

It is important to note that other imaging approaches could yield useful, complementary information. Therefore, a larger scale study with more patients, different levels of damage to cortex and using a broader set of imaging modalities based on MR or MEG (see e.g., Kupers et al., 2021) would be very timely. The emphasis of this paper is to establish methodologies for identifying and clearly defining parts of the post-stroke brain that retain the potential to support some usable visual function.

## Data Availability Statement

The datasets presented in this study can be found in the following online repository: https://gin.g-node.org/schluppeck/strkvis-mri.

## Ethics Statement

The studies involving human participants were reviewed and approved by the University of Nottingham, School of Psychology Ethics Committee (F944/F1055R). The patients/participants provided their written informed consent to participate in this study.

## Author Contributions

AB, BW, and DS designed the experiments and collected data for this study. AB, PM, and DS wrote the manuscript. All authors contributed to the analysis and interpretation of data.

## Conflict of Interest

The authors declare that the research was conducted in the absence of any commercial or financial relationships that could be construed as a potential conflict of interest.

## Publisher’s Note

All claims expressed in this article are solely those of the authors and do not necessarily represent those of their affiliated organizations, or those of the publisher, the editors and the reviewers. Any product that may be evaluated in this article, or claim that may be made by its manufacturer, is not guaranteed or endorsed by the publisher.
