# Supplementary material for: Linking Multi-Modal MRI to Clinical Measures of Visual Field Loss After Stroke
Source: Front Neurosci. 2022 Jan 5;15:737215. doi: 10.3389/fnins.2021.737215 (PMC8766758; doi:10.3389/fnins.2021.737215)
Supplement: Supplementary file 1 [file Data_Sheet_1.pdf]

Supplementary Table 1: Results of optometric assessment

Subject	Visual Field Loss	Eye	Refractive Status	Distance VA (logMAR)	Near Addition	Near VA
11773	Hemianopia	R	-3.25	-	-	-
	Hemianopia	L	-3.25	-	-	-
13978	Quandrantanopia	R	+1 / -0.75 @100	-0.06	2.25	0.6
	Quandrantanopia	L	+1.25 / -0.5 @62.5	-0.08	2.25	1.0
14196	Hemianopia	R	+2.5 / -0.75 @25	-0.10	2.00	0.5
	Hemianopia	L	2.75 / -0.25 @175	-0.20	2.25	0.1
14326	Quandrantanopia	R	+1.5 / -2.00 @115	0.10	2.50	0.3
	Quandrantanopia	L	+1.5 / -2.00 @75	0.0	2.50	0.1

For all participants in the study (for each eye), no movement was detected during a *cover test, ocular motility* was normal, *pupillary responses* were normal, no abnormalities were detected neither during an *internal exam* nor an *external exam,* and there was no evidence of *spatial neglect.*

Appendix A

Brief Description from Participant debriefs

11773: Experienced a stroke in late 20s. Reported a dense hemianopia in the right visual field. Reported difficulties with every-day tasks.

13798: Experienced a stroke early August 2018 and after some level of initial spontaneous recovery was left with distorted vision in the lower left quadrant of the visual field. Reported difficulty in reading the newspaper/books, used a ruler as a guide to help find the next line of text. This is also the only participant who reported rare visual hallucinations in the 'blind' field, but only when extremely tired.

14196: Experienced a stroke roughly 25 years ago. Reports total dense visual loss in right visual field. Also affected by aphasia.

14326, Experienced a stroke July 2016 and reports visual field loss in lower left quadrant. Was able to regain drivers license after follow-up measurements of his visual impairment.

Appendix B

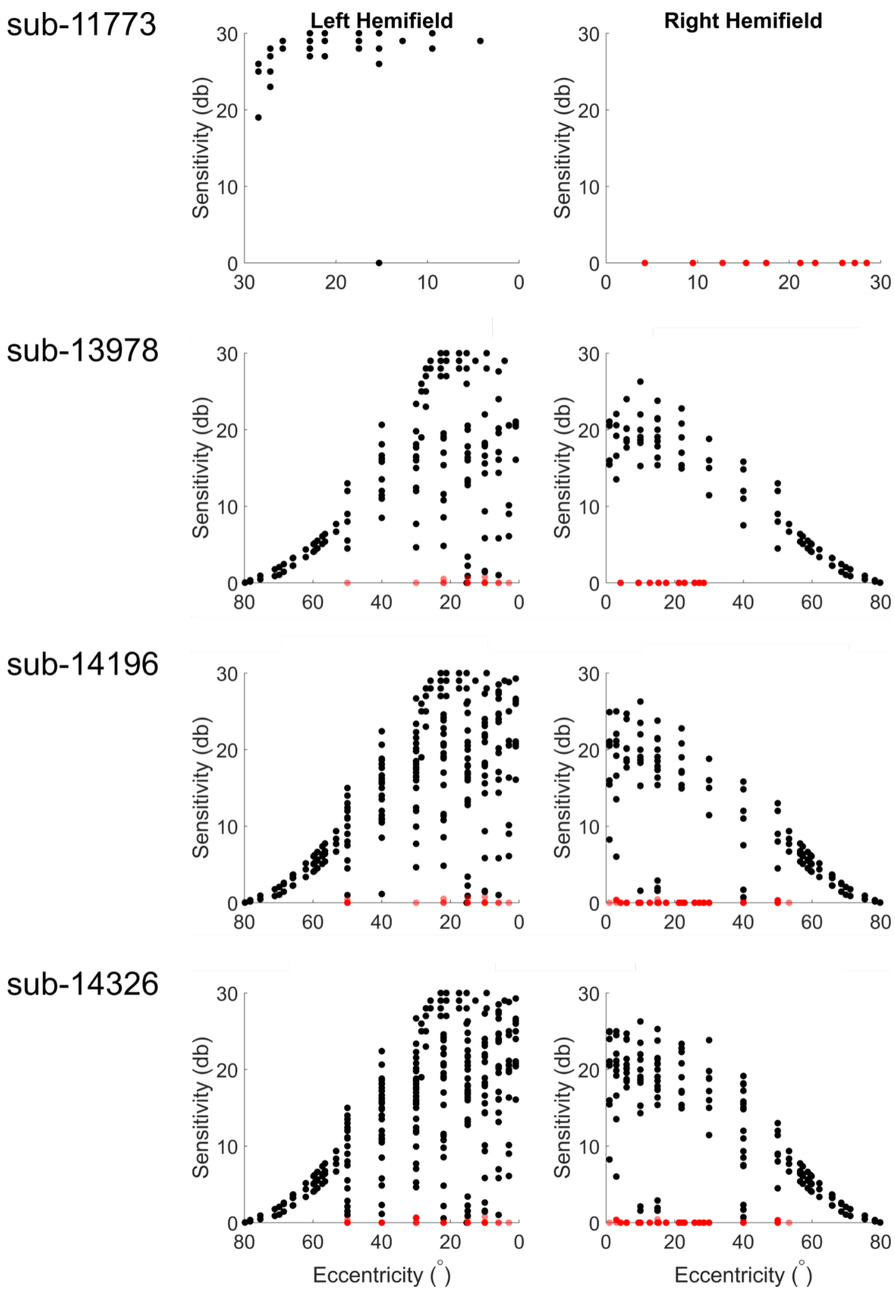

Supplementary Figure 1

Sensitivity measurements from static perimetry test for all four participants. Scatter plots show sensitivity (db) as a function of eccentricity for all test points in perimetry, separately for left and right hemifields. Red symbols represent test points that fall below threshold for vision (adjusted for age) and correspond to points in the scotoma. They are all at ~0 dB (and points at the same eccentricity may be overplotted here). Note that perimetry for sub-11773 covered a more central region of test points only.

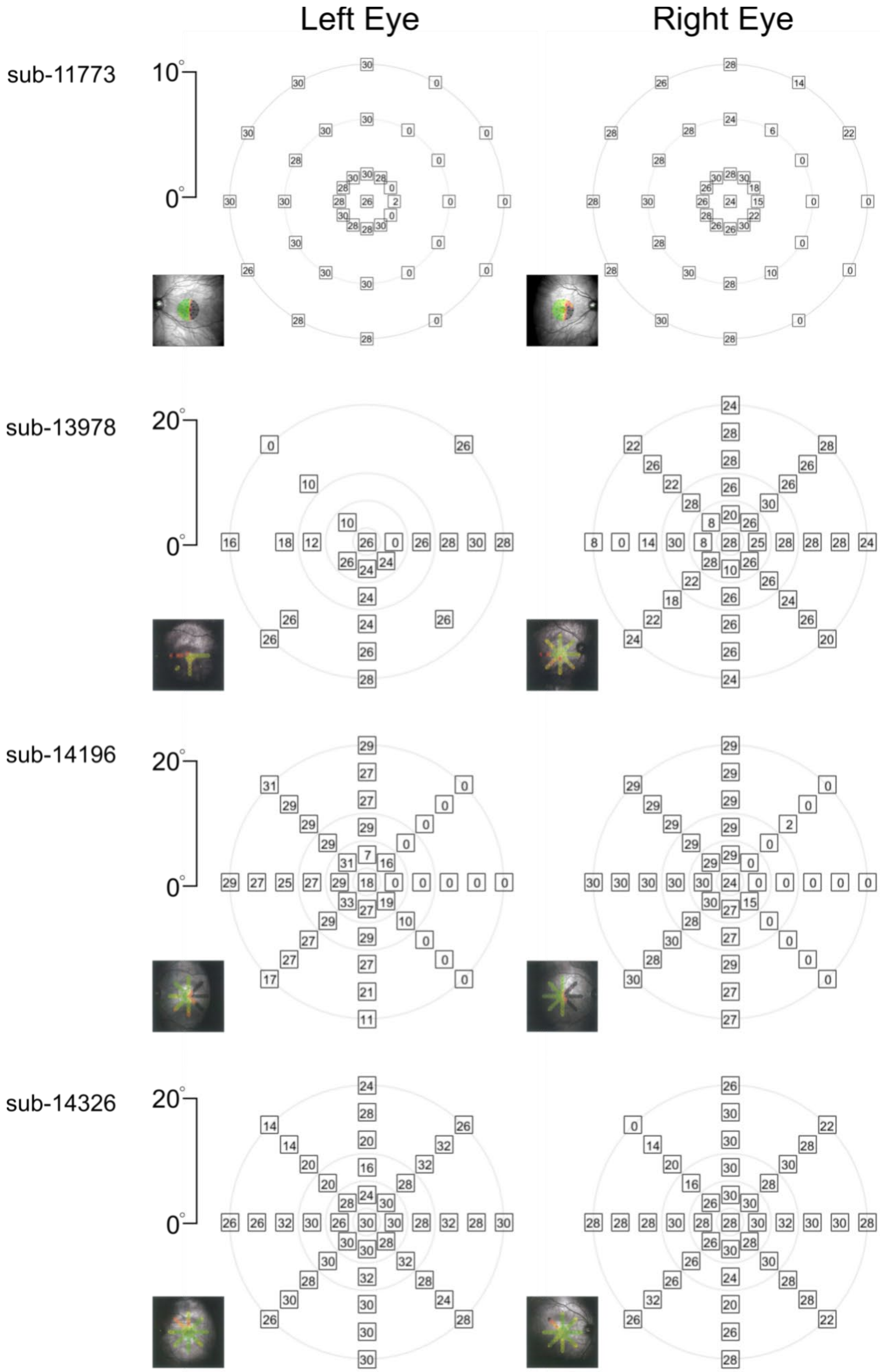
Left Eye
Right Eye
sub-11773
10°
0°
sub-13978
20°
0°
sub-14196
20°
0°
sub-14326
20°
0°

Supplementary Figure 2

Microperimetry measurements across 4 stroke survivors. The measurements across the sample points represent the retinal sensitivity (db). All stroke survivors were measured using a 41-point stimulus grid which spans 20° with the exception of participant 11773 which uses a 37-point stimulus grid which spans 10° (a subsequent stroke in this participant prevented us from acquiring visual field maps with exactly matched sampling points). Note that for participant 13978, fixation stability was an issue. Even with gaze-contingent microperimetry, acquiring robust data was challenging. For participant 14326, robust macular sparing was apparent in both standard and microperimetry (see Figure 2). Despite this, the fMRI measurements still reveal a different pattern of loss (cf Table 3, and Figure 5).
